# Triangular-Shaped Odontoid Process With Chiari 1 Malformation Patient

**DOI:** 10.7759/cureus.10788

**Published:** 2020-10-04

**Authors:** Katherine Cironi, Joe Iwanaga, Aaron S Dumont, R. Shane Tubbs

**Affiliations:** 1 Department of Neurosurgery, Tulane Center for Clinical Neurosciences, Tulane University School of Medicine, New Orleans, USA; 2 Neurosurgery and Ochsner Neuroscience Institute, Ochsner Health System, New Orleans, USA; 3 Department of Anatomical Sciences, St. George’s University, St. George's, GRD

**Keywords:** chiari 1 malformation, odontoid process, morphology, axis, imaging

## Abstract

Several anatomical variations of osseous structures around the craniovertebral junction (CVJ) have been observed in those presenting with Chiari 1 malformation (CM-1) due to the junction’s unique embryology and its pivotal role in neck stability. During a clinic visit, a 14-year-old female presented with the classic symptoms of CM-1. Upon follow-up imaging and confirmation of the inferiorly displaced cerebellar tonsils, a unique triangular-shaped odontoid process was identified. To our knowledge, this osseous malformation of the dens has not been reported in the current literature. This unique deviation may cause ligamentous instability and decreased motion capacity and predispose a patient to axial fractures. Thus, we aim to further discuss this case, cervical vertebrae axis (C2) embryology, and the resulting clinical significance of this observed odontoid process variant.

## Introduction

Chiari 1 malformation (CM-1) is defined as the caudal displacement of the cerebellar tonsils downward through the foramen magnum, more than 5 mm below McRae’s line [1]. Osseous malformations in the craniocervical junction have been found in about 50% of patients diagnosed with CM-1 [2]. Due to the unique secondary ossification center in the apical segment of the odontoid process, there is potential for variant architectural growth. Odontoid process angulation has been noted to be associated with increased tonsillar ectopia [3]. Regardless, in normal patients and patients with CM-1, the odontoid process is peg-shaped [4]. Here, we present a rare variant of the apical part of the odontoid process in a patient with CM-1. Bringing attention to such an anatomical variation might aid the neurosurgeon, head and neck surgeon, radiologist, pediatrician, and neurologist in diagnosing and understanding unique osseous findings in patients with this type of hindbrain herniation.

## Case presentation

**Figure 1:**
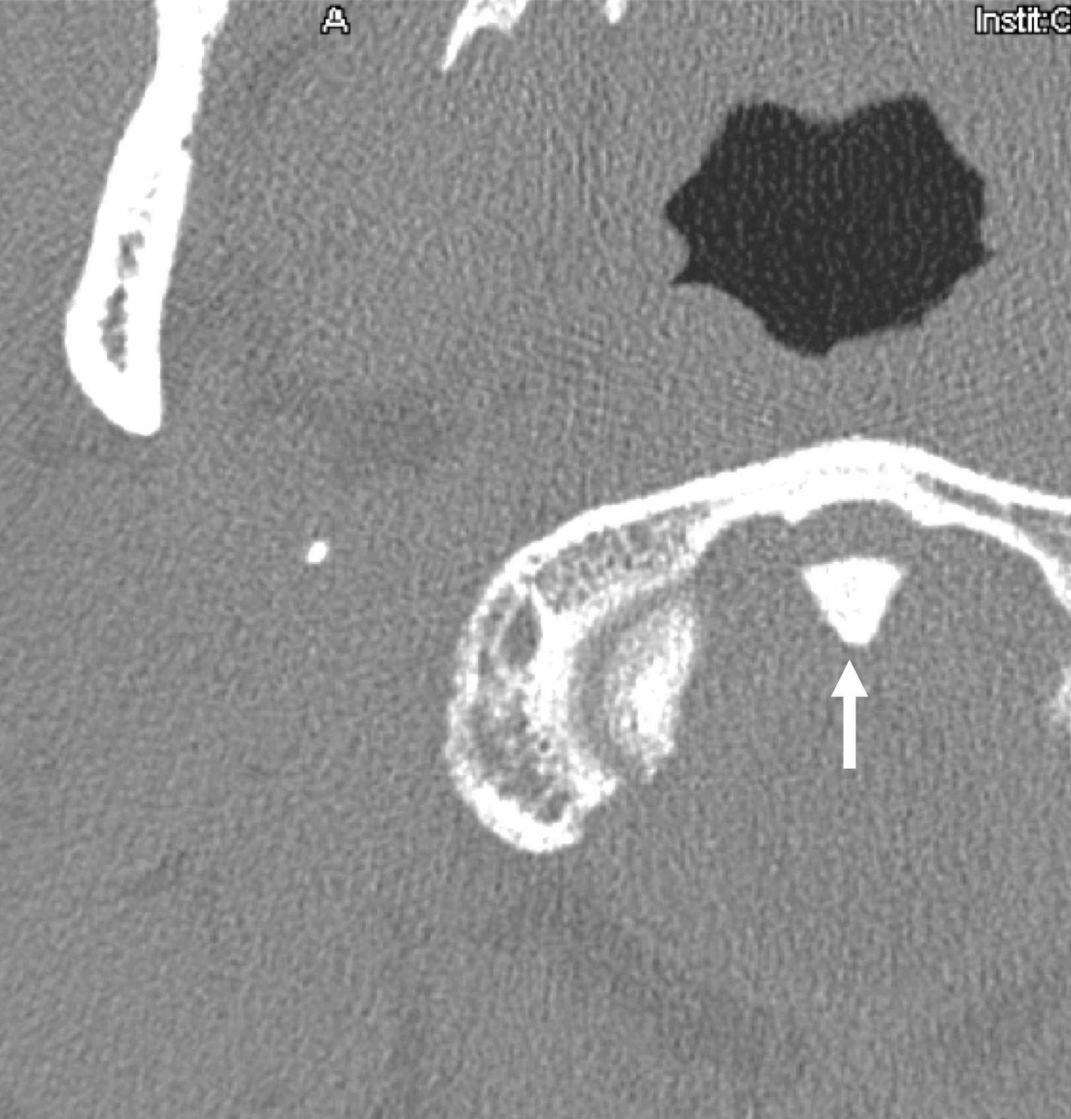
Axial CT of the skull in the case Note the triangular-shaped odontoid process (arrow). CT: computed tomography

**Figure 2:**
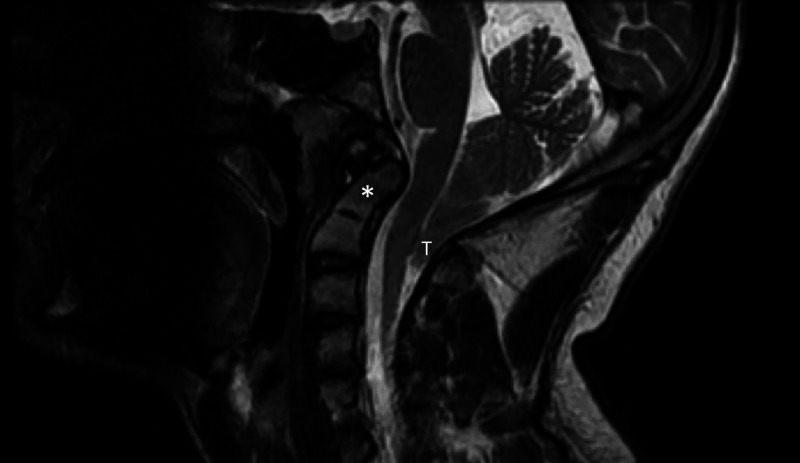
T2-weighted, sagittal MRI Note the herniated cerebellar tonsils (T) indicate a CM-1 and the retroverted odontoid process (asterisk). MRI: magnetic resonance imaging, CM-1: Chiari 1 malformation

A 14-year-old female patient presented to our clinic with a Valsalva-induced headache. Magnetic resonance imaging (MRI) ordered by her pediatrician diagnosed a CM-1. Her headaches occurred three to four times per week and involved the occipital region only. The pain did not radiate beyond the vertex of the skull. Her neurological examination was within normal limits. There was no pain with range of motion of the neck. The CM-1 was found to have 6-7 mm of caudal herniation of the cerebellar tonsils below the foramen magnum. There was no hydrocephalus or syringomyelia. MRI suggested atlanto-occipital fusion. Therefore, computed tomography (CT) of the neck and craniocervical junction was performed, which identified a triangularly shaped odontoid process (Figure 1) and showed herniated cerebellar tonsils (Figure 2).

The length of the process was felt to be within normal limits but the width was shorter than normal at 7.5 mm. No other bony variants, e.g., basilar invagination, were noted, including the suspected atlanto-occipital assimilation. The patient underwent a standard posterior fossa decompression procedure with duraplasty. No complications occurred due to the procedure, and at the one-week follow-up, the wound was healing well and the patient had no complaints of the preoperative Valsalva-induced headache. At the one-year follow-up, the patient continued to be free of her preoperative symptoms.

## Discussion

Projecting superiorly through the atlas, the odontoid process is of critical importance to the craniovertebral junction (CVJ), with slight variations causing potential instability at the median atlantoaxial joint [5]. Embryologically, the odontoid process is traditionally said to originate from the anterior atlas and then fuses caudally with the body of the axis at the sixth to seventh week of gestation [6-7]. However, these details have been disputed. In general, the odontoid process is formed via two laterally separated primary ossification centers that fuse in the midline by the seventh to eighth month in utero [8]. The midline unified primary ossification center proceeds to grow apart from the body of the axis until its fusion between three and six years of life. This coalescence is termed the subdental synchondrosis [6,9]. A cause of the abnormally-shaped odontoid process in our patient may be the growth of the apex being distinct from the odontoid body. This apical secondary ossification center fuses with the remainder of the odontoid process by 12 years of age [6,9-10]. The presented case most likely had an abnormal bone growth at the secondary ossification center, causing this anatomical malformation and potentially contributing to CM-1 symptoms, as the triangular-shaped odontoid process protruded further posteriorly than normal.

The variant bone development of the CVJ has been well-studied and is commonly seen in patients with CM-1. First analyzed by Tubbs et al. [10] and then validated by Ladner et al. [3], some degree of retroflexion of the odontoid process is found in over 80% of CM-1 patients; furthermore, the odontoid process continues to grow posteriorly with age in this group. This unique growth pattern can cause further compression of the spinal canal, which is already compromised due to the ectopically herniated cerebellar tonsils. The correlation with angulation and increasing tonsillar ectopia should be further studied, as Tubbs et al. [10] found that a posteriorly inclined odontoid process is not correlated with worsening CM-1 symptoms, but higher grades of angulation can be associated with a greater chance of developing syringomyelia. On the contrary, Ladner et al. [3] concluded that more posteriorly displaced cerebellar tonsils were correlated with increasing retroflexion of the odontoid process but no relationship with syringomyelia was noted. Due to the unique structure of this CM-1 patient’s odontoid process, the angle of the apex has the potential to further impinge on the downwardly displaced cerebellar tonsils.

The triangular-shaped odontoid process reported here is both thinner and more angled than normal. The mean width of a normal odontoid process is about 11 mm [8,10], whereas our case’s width at its midpoint was only 7.5 mm. The triangular-shaped odontoid process may reduce the stability of the CVJ due to the diminished surface area for its surrounding ligaments to attach. The two alar ligaments have lateral attachments on the odontoid process and insert on the medial aspect of the occipital condyle; between these is the apical ligament, inserting from the apex of the odontoid into the anterior foramen magnum [11-12]. Sardi et al. found 16 of the 22 sides to have alar ligaments with posterolateral attachments on the top half of the odontoid process, extending up to the apex [12]. Our misshaped dens may alter the attachment of the alar ligaments and inhibit their main role - to restrict cervical rotation and bending to the contralateral side. Any lesion or tear of these alar ligaments could cause an increased range of motion, potentially leading to occipitocervical instability [12]. Restraining the neck of the odontoid process, the transverse ligament attaches on either side of the transverse ligament tubercles of the atlas. As the strongest of the three, it further constricts the atlantoaxial joint from excessive movement [9]. Karaaslan et al. [13] examined the MRIs of 25 adults with CM-1 and compared those to 93 healthy adult subjects. They found CM-1 patients have significantly shorter transverse and alar ligaments when compared to the normal population, speculating that ligamentous variations may be a significant cause of disease etiology or progression [13]. Although not substantiated, our variant odontoid process may play a role in further diminished ligamentous stability and motion capacity in the atlas-axial joint in this CM-1 patient. Lastly, a shorter width of the odontoid process might predispose an individual to fractures of the odontoid process [14].

## Conclusions

As variations of the odontoid process can lead to pathology, knowledge of a triangularly shaped process is important. The angulation of the dens noted in this patient with CM-1 may predispose their cerebellar tonsils to further impingement and reduce the stability of the craniocervical junction. This malformation could play a factor in her Valsalva-induced headaches and affect her range of motion in the next few decades of life. Therefore, those clinicians who view imaging of the craniocervical junction should be aware of such varied morphology of the axis.
